# Waterpipe smoking and the risk of myocardial infarction: A hospital-based case-control study

**DOI:** 10.18332/tid/114074

**Published:** 2019-12-02

**Authors:** Abdulmajeed Al-Amri, Khalid Ghalilah, Assaf Al-Harbi, Sami A.R. Al-Dubai, Saleh Al-Ghamdi, Abdulmohsen Al-Zalabani

**Affiliations:** 1 Public Health Department, Ministry of Health, Al-Madinah, Kingdom of Saudi Arabia; 2 Internal Medicine Department, Al-Madinah Specialist Hospital, Al-Madinah, Kingdom of Saudi Arabia; 3 Department of Dermatology, Ohud Hospital, Al-Madinah, Kingdom of Saudi Arabia; 4 Joint Program of Family Medicine, Ministry of Health, Al-Madinah, Kingdom of Saudi Arabia; 5 Saudi Board of Preventive Medicine, Ministry of Health, Al-Madinah, Kingdom of Saudi Arabia; 6 Department of Cardiology, King Faisal Specialist Hospital & Research Center, Jeddah, Kingdom of Saudi Arabia; 7 Department of Family and Community Medicine, College of Medicine, Taibah University, Al-Madinah, Kingdom of Saudi Arabia

**Keywords:** waterpipe smoking, myocardial infarction, shisha, hookah, risk factors

## Abstract

**INTRODUCTION:**

The prevalence of waterpipe smoking is increasing globally. The involvement of waterpipe smoking as an independent risk factor for the development of myocardial infarction is an area in need of further research. Our study examines the possible relationship between waterpipe smoking and myocardial infarction.

**METHODS:**

In this incident case-control study, we enrolled 148 cases with myocardial infarction and 148 participants in the control group. Using logistic regression models, odds ratios and 95% CIs were calculated for the association between waterpipe smoking and myocardial infarction, while adjusting for possible confounders.

**RESULTS:**

Myocardial infarction was associated significantly with current waterpipe smoking (OR=4.08; 95% CI: 1.37–12.10), ever waterpipe smoking (OR=3.6; 95% CI: 1.31–10.19), and exclusive waterpipe smoking (OR=10.26; 95% CI: 2.22–47.29). Exclusive cigarette smoking was also associated significantly with the development of myocardial infarction (OR=4.6; 95% CI: 1.98–11.04).

**CONCLUSIONS:**

Waterpipe smoking is associated with myocardial infarction. Our findings reveal the need for targeted interventions to reduce the prevalence of this globally spreading form of tobacco smoking.

## INTRODUCTION

Waterpipe smoking (also known as shisha, hookah, and narghile) is a recent, global tobacco epidemic, especially among youths^1^. Factors associated with this accelerating trend have been investigated in a recent review that concluded that social acceptance, missing or weak laws, positive views toward waterpipe smoking, false beliefs about the lack of addictive potential, and ease of access, were the main contributing factors^2^. Nevertheless, waterpipe smoking is associated with many health events. Waterpipe exposure has been linked to cancer, including cancer of the head and neck, esophageal cancer, stomach cancer, lung cancer, and bladder cancer^3^. Waterpipe smoking is also associated with chronic obstructive pulmonary disease, bronchitis, metabolic syndrome, and low birth weight^4^. The relationship between waterpipe smoking and myocardial infarction as a distinct, independent risk factor is still in need of further research. Previous studies on waterpipe smoking have answered some of the research questions, as a challenge remains the separation of the effect of cigarette smoking alone from that of waterpipe smoking, as dual use is very common^5,6^, while other studies, such as the study by Platt et al.^7^ provided interesting results with different study designs. A recent statement from the American Heart Association indicated the need for epidemiological research on the possible link between waterpipe smoking and cardiovascular diseases, especially in regions where waterpipe smoking is more prevalent^8^. Indirect evidence of a possible association is indicated by the fact that a single waterpipe smoking session is associated with more carbon monoxide and smoke exposure and similar nicotine exposure compared to smoking one cigarette^9^. In this study, we aimed to investigate the possible relationship between waterpipe smoking and myocardial infarction.

## METHODS

### Design, settings and participants

We used an incident, hospital-based non-matched case-control study design, which was performed in Al-Madinah from January 2017 to February 2018. Al-Madinah city is located in the western part of Saudi Arabia, with an estimated population of 2 million. The health care system in Saudi Arabia enables free and full access to health care services for all citizens, with the Ministry of Health serving as the major healthcare provider. This study followed the Strengthening the Reporting of Observational Studies in Epidemiology (STROBE) reporting guideline^10^.

#### Selection of cases

Cases were recruited from Al-Madinah Cardiac Center, which is a specialized centre for patients with heart diseases. It has a capacity of 100 beds in addition to 34 beds for intensive cardiac care. It was chosen as the source of cases because it is the main centre in the region for referral and treatment of myocardial infarction patients. All available myocardial infarction cases diagnosed from January 2017 to February 2018 were assessed for eligibility. Inclusion criteria included being an incident case of myocardial infarction, a first-time diagnosis of myocardial infarction, and patients meeting the case definition. Exclusion criteria included cases of myocardial infarction with those aged <18 years, non-atherosclerotic causes of myocardial infarction, unstable angina, cases of myocardial infarction with significant chronic comorbidities (liver diseases, renal diseases, and malignancies), and cases of myocardial infarction referred from outside Al-Madinah city.

#### Selection of controls

Controls were recruited from the Dermatology and the Surgery departments in Ohud Hospital, which currently has a bed capacity of 261. It is considered a referral centre for the dermatology and surgery specialties, providing services for the same population covered by Al-Madinah Cardiac Center, which makes it a very suitable source of controls in this study. Inclusion criteria for the control group included patients with skin or surgical disorders not related to the exposure of interest. Examples of such disorders that have no apparent link to tobacco smoking include patients diagnosed with acne vulgaris, hair loss, abscess drainage, urticaria, pilonidal sinus, and hemorrhoids. Exclusion criteria for the control group included patients aged <18 years, patients with significant chronic comorbidities (liver diseases, renal diseases, and malignancies), history of heart diseases, history of angina, and patients referred from outside the city. In addition, patients with outcomes that have an association with tobacco smoking were excluded (e.g. inguinal hernia, appendicitis, and psoriasis).

### Variables and data measurement

A structured questionnaire and medical records were used as data sources. A phone interview was conducted for each case of myocardial infarction because most of the participants with myocardial infarction were discharged from the cardiac centre at the time of data collection. Face-to-face interviews were conducted for the participants in the control group. Data were collected by trained interviewers. Medical records were used to obtain data on height, weight, and lipid profile. Fasting blood samples were drawn from the surgical patients at admission, and from the dermatology patients at the outpatient clinic laboratory. Fasting blood samples were drawn for cases of myocardial infarction within 24 hours of admissions. The first blood measurement for lipids was considered, and any subsequent measurements were excluded to avoid any alteration in the blood lipids level due to medical interventions.

The outcome of interest was myocardial infarction. The third universal definition of myocardial infarction was used^11^. Briefly, a diagnosis of myocardial infarction was made if an abnormal level of cardiac troponin was detected. In addition, one of the following had to be present: characteristic ECG changes, symptoms of ischemia, or echocardiographic evidence.

The exposure of interest was waterpipe smoking. Waterpipe smoking was defined according to the ‘consensus statement on assessment of waterpipe smoking in epidemiological studies’^12^, in which a current waterpipe smoker was defined as smoking a waterpipe at least once in the past month. Ever waterpipe smoking was defined as ever engaging in a waterpipe smoking session. To examine the specific effects of waterpipe smoking, an exclusive waterpipe smoking variable was computed by excluding cigarette smokers and cigarette-waterpipe dual-users. Furthermore, an exclusive cigarette smoking and cigarette-waterpipe dual smoking variables were computed in order to compare the combined and separate effects of both forms of tobacco smoking. Current cigarette smoker was defined as smoking cigarettes in the past 12 months^13^.

Other variables included the history of medical diagnosis of diabetes mellitus and hypertension, physical activity, and obesity. Physical activity was defined as participation in moderate exercise for 150 minutes or more per week or participation in strenuous exercises for 75 minutes or more per week^14^. Obesity was defined as a body mass index of ≥30 kg/m^2^. Blood levels of cholesterol, low-density lipoprotein, high-density lipoprotein, and triglycerides were obtained for all participants from their medical records. Hypercholesterolemia was defined as a cholesterol level above 6.1 mmol/L. Hypertriglyceridemia was defined as a triglyceride level above 2.28 mmol/L. Low levels of high-density lipoprotein were defined as levels below 1.04 mmol/L. High levels of low-density lipoprotein were defined as levels above 2.3 mmol/L. The following variables were defined as per the INTERHEART study methodology^13^: stress at home, stress at work, financial stress, and stressful life events. Briefly, stress was defined as feeling irritable, filled with anxiety or having sleeping difficulties as a result of conditions at work or at home. Stress had four levels: none, some periods, several periods or permanent stress. Financial stress had three levels: little or none, moderate or severe financial stress. Stressful life events include history of one or more of the following events: marital separation or divorce, loss of job or retirement, business failure, violence, major family conflict, major personal injury, death or major illness of a close family member, the death of a spouse or other major stress^13^. All participants with myocardial infarction were asked to respond to the questionnaire about their conditions prior to their myocardial infarction diagnosis.

### Sample size

Based on a previous national survey^15^, the prevalence of waterpipe smoking in the control group was assumed to be 12.44%. A hypothetical odds ratio (OR) of 2.4 for waterpipe smoking in cases of myocardial infarction was assumed based on the literature^16^. With 80% power and a 5% significance level, the calculated sample size was 143 cases and 143 controls. The sample size was calculated using the formula in the method described in Kelsey et al.^17^.

### Ethical considerations

Informed consent was obtained from all participants. The ethical committee of the Directorate of Health in Al-Madinah approved the study protocol. The participants were informed about the purpose of this study, the expected risks and benefits, and that participation was voluntary. All information obtained from the participants was treated in a confidential matter.

### Statistical analysis

Statistical analysis was performed using SPSS version 23 (IBM, Armonk, New York). Chi-squared and Student’s t-test were used to compare the baseline characteristics between cases and controls. Univariate and multivariable logistic regression was used to test the independent association between waterpipe smoking and myocardial infarction, adjusting for possible confounders. In addition to adjusting for recognized confounders such as age, sex, and cigarette smoking, we used a change-in-estimate criterion to identify possible confounders. Any variable that altered the waterpipe-myocardial infarction effect by 10% was identified as a possible confounder. The final multivariable logistic regression models were adjusted for age, sex, hypertension, diabetes, cigarette smoking, high cholesterol and high low-density lipoprotein (LDL).

## RESULTS

There were 148 cases in the myocardial infarction group and 148 participants in the control group. In total, 35 participants (11.8%) were current waterpipe smokers. Among these current waterpipe smokers, 25 (71.4%) were current daily smokers, while the remainder were non-daily waterpipe current smokers. The average duration of waterpipe smoking was 24 ± 2.1 years, while the average days of smoking waterpipe per month were 21 ± 1.8 days. The average sessions smoked per month were 41 ± 6 sessions. In current waterpipe smokers, 18 participants (46.2%) smoked waterpipe at public places, 16 participants (41%) smoked waterpipe at home, and 5 participants (12.8%) smoked waterpipe at friends’ homes.

Participants who developed myocardial infarction were significantly older males (mean ± SD: 54.1 ± 10.7 years in cases vs 41.6 ± 15.3 years in controls, p< 0.001). Cases were more likely to have a history of diabetes (58.8% vs 12.2%; p<0.001), hypertension (60.1% vs 15.5%; p< 0.001), cigarette smoking (41.2% vs 18.9%; p<0.001), high cholesterol (18.9% vs 4.1%; p<0.001), high LDL (25.7% vs 6.8%; p<0.001), and low HDL (48% vs 32.4%; p=0.004) (Table 1).

**Table 1:** Baseline characteristics of incident myocardial infarction cases and unmatched controls, Al-Madinah, Saudi Arabia (N=296)

*Characteristics*	*Cases n (%)*	*Control n (%)*	*OR ( 95% CI)*	*p*
Age (years), mean (SD)	54.1 (10.7)	41.6 (15.3)		<0.001
Gender (male)	123 (83.1)	80 (54.1)	4.18 (2.44–7.16)	<0.001
Marital status (married)	129 (87.2)	105 (70.9)	2.78 (1.52–5.05)	<0.001
Employment status (unemployed)	40 (27)	67 (45.3)	2.23 (1.37–3.63)	<0.001
Obesity	47 (31.8)	41 (27.7)	0.82 (0.5–1.38)	0.261
Cigarette smoking	61 (41.2)	28 (18.9)	3 (1.77–5.08)	<0.001
Current waterpipe smoking	28 (18.9)	7 (4.7)	4.7 (1.98–11.14)	<0.001
Hypertension	89 (60.1)	23 (15.5)	8.19 (4.71–14.25)	<0.001
Diabetes	87 (58.8)	18 (12.2)	10.3 (5.7–18.61)	<0.001
Hypercholesterolemia	28 (18.9)	6 (4.1)	5.52 (2.21–13.78)	<0.001
Low HDL	71 (48)	48 (32.4)	1.92 (1.19–3.07)	0.004
High LDL	38 (25.7)	10 (6.8)	4.76 (2.27–9.9)	<0.001
Hypertriglyceridemia	21 (14.2)	15 (10.1)	1.46 (0.72–2.97)	0.181
Education (university vs none)	32 (21.6)	56 (36.5)	0.48 (0.28–0.80)	0.003
Stress at work (permanent)	9 (6.1)	1 (0.7)	9.51 (1.19–76.1)	<0.001
Stress at home (permanent)	13 (8.8)	3 (2)	4.65 (1.29–16.69)	0.009
Financial stress (severe)	27 (18.2)	8 (5.4)	3.9 (1.71–8.91)	<0.001

SD: standard deviation, HDL: high-density lipoprotein, LDL: low-density lipoprotein.

In the univariate analysis, current waterpipe smoking was associated with increased odds of myocardial infarction (OR=4.7; 95% CI: 1.98–11.14). In the multivariable logistic regression model adjusted for age and sex (Model 1), current waterpipe smoking was still associated with increased odds of myocardial infarction (OR=3.03; 95% CI: 1.1–7.8). After further adjustment for the covariates (Model 2), current waterpipe smoking was associated with even higher odds of myocardial infarction (OR=4.08; 95% CI: 1.37–12.10). A similar significant association was observed for ever waterpipe smoking (OR=3.6; 95% CI: 1.31–10.19) (Table 2). Exclusive cigarette smoking and exclusive waterpipe smoking were associated significantly with the development of myocardial infarction (OR=4.6; 95% CI: 1.98–11.04 and OR=10.26; 95% CI: 2.22–47.29, respectively). On the other hand, cigarette-waterpipe dual smoking was associated with myocardial infarction (OR=2.57) but the association was not statistically significant (95% CI: 0.51–12.88) (Table 3).

**Table 2:** Multivariate analysis for the association between waterpipe smoking and myocardial infarction, Al-Madinah, Saudi Arabia (N=296)

*Variables*	*OR crude ( 95% CI)*	OR adjusted* (95% CI) (Model 1)	OR adjusted** (95% CI) (Model 2 )
Current waterpipe smoking	4.7 (1.98–11.14)	3.03 (1.1–7.8)	4.08 (1.37–12.10)
Ever waterpipe smoking	4.6 (2.05–10.4)	2.82 (1.15–6.92)	3.6 (1.31–10.19)

OR: odds ratio, CI: confidence interval, LDL: low-density lipoprotein.

* Adjusted for age and sex.

** Adjusted for age, sex, hypertension, diabetes, cigarette smoking, high cholesterol and high LDL.

**Table 3:** Multivariate analysis for the association between exclusive cigarette smoking, exclusive waterpipe smoking and myocardial infarction, Al-Madinah, Saudi Arabia (N=296)

*Variables*	*OR crude ( 95% CI)*	OR adjusted* (95% CI)
Exclusive cigarette smoking	3.8 (2.18–6.80)	4.6 (1.98–11.04)
Exclusive waterpipe smoking	11.64 (3.33–40.63)	10.26 (2.22–47.29)
Dual smoking cigarette + waterpipe	3.49 (1.01–12.03)	2.57 (0.51–12.88)

OR: odds ratio, CI: confidence interval, LDL: low-density lipoprotein.

* Adjusted for age, sex, hypertension, diabetes, high cholesterol and high LDL.

## DISCUSSION

Our findings indicated that waterpipe smoking was associated significantly with the occurrence of myocardial infarction. To our knowledge, this case-control study is one of the earliest that specifically designed and executed an epidemiologic study to better understand the relationship between waterpipe smoking and cardiovascular diseases.

Our findings support the preliminary results of previous studies. A previous case-control study found that waterpipe smoking was associated with coronary heart disease, although the association was not statistically significant^16^. In another observational study conducted in Egypt among patients who underwent coronary angiography, waterpipe smoking was associated with more severe coronary artery disease compared to non-smokers, cigarette smokers, and mixed smokers. However, the results of that study were limited by the lack of effect estimates^5^. Another study in Lebanon found that in 1210 participants who underwent coronary angiography, cumulative waterpipe smoking (waterpipe-years) was associated with a more severe coronary artery stenosis compared to non-smokers. Nevertheless, waterpipe smoking status in that study did not appear to be associated with coronary artery disease^6^. In a recent similar study that was also conducted in Lebanon, waterpipe smoking was associated significantly with myocardial infarction in catheterized patients^7^. A recent cross-sectional, community-based study conducted in Qatar and Lebanon found that, compared to non-smokers, exclusive waterpipe smoking was associated significantly with increased risk of long-term occurrence of coronary artery disease, as indicated by the presence of calcification on arterial walls, which was assessed by using multidetector computed tomography^18^.

The exact mechanism by which tobacco induces atherosclerosis and subsequent ischemia is not fully understood. Oxidative stress and endothelial dysfunction have been proposed as a possible underlying mechanism^19,20^. A previous study found that brachial artery flow-mediated vasodilation was significantly impaired among waterpipe smokers compared to cigarette smokers and non-smokers, which demonstrates the involvement of waterpipe-derived toxins in endothelial dysfunction^21^. Waterpipe smoking has been previously associated with oxidative injury^22^. Direct comparisons of the chemical constituents of cigarette smoke and waterpipe smoke showed that waterpipe smokers and passive waterpipe smokers are exposed to higher doses of particulate matter, polycyclic aromatic hydrocarbons, and volatile aldehydes benzene^23,24^. Previous studies show that waterpipe smoking meets the WHO definition for nicotine dependence^25,26^. This combination of high chemical exposure and dependence leaves waterpipe smokers at high risk for many adverse health events including myocardial infarction. Even though the pathophysiology of tobacco-induced atherosclerosis remains largely unknown, smoking cessation continues to be effective both at the primary prevention level and in patients with established ischemic heart disease^27,28^.

## Strengths and limitations

One of the strengths of this study is the use of case-control study design, as most previous studies that addressed the health effects of waterpipe smoking were cross-sectional. Other strengths include using standardized questionnaires and inclusion of cases with a first-time diagnosis of myocardial infarction. To minimize the risk of recall bias, we included only incident cases of myocardial infarction. To minimize the risk of selection bias, we excluded all referred patients from outside the city in both the case and control groups. Furthermore, we selected participants in the control group with different diagnoses to decrease the effect of selecting controls with a disease that has a hidden relationship with the exposure of interest. Some limitations in this work should also be noted. First, diabetes mellitus and hypertension were coded based on patients’ self-report. Second, we had a small sample, leading to less precise estimates. Given the case-control design of this study, the association we found may or may not represent a causal relationship. In addition, the effects of unknown confounders cannot be excluded.

## CONCLUSIONS

Our results indicate that waterpipe smoking is a risk factor for the development of myocardial infarction. Our findings indicate the need for a change in the laws and regulations toward waterpipe smoking and modifications in the practice of health education and smoking cessation programs. Further research, especially the use of longitudinal studies, is needed to confirm our findings. In addition, the association between waterpipe smoking and other health outcomes, such as stroke and lung cancer, deserves special attention.
